# The Double Mutation *DSG2*-p.S363X and *TBX20*-p.D278X Is Associated with Left Ventricular Non-Compaction Cardiomyopathy: Case Report

**DOI:** 10.3390/ijms22136775

**Published:** 2021-06-24

**Authors:** Roman Myasnikov, Andreas Brodehl, Alexey Meshkov, Olga Kulikova, Anna Kiseleva, Greta Marie Pohl, Evgeniia Sotnikova, Mikhail Divashuk, Marina Klimushina, Anastasia Zharikova, Maria Pokrovskaya, Sergey Koretskiy, Maria Kharlap, Elena Mershina, Valentin Sinitsyn, Elena Basargina, Leila Gandaeva, Vladimir Barskiy, Sergey Boytsov, Hendrik Milting, Oxana Drapkina

**Affiliations:** 1 National Research Center for Therapy and Preventive Medicine, Petroverigskiy Lane 10, 101990 Moscow, Russia; andorom@yandex.ru (R.M.); meshkov@lipidclinic.ru (A.M.); olgakulikova2014@mail.ru (O.K.); sotnikova.evgeniya@gmail.com (E.S.); divashuk@gmail.com (M.D.); mklimushina@gmail.com (M.K.); azharikova89@gmail.com (A.Z.); MPokrovskaya@gnicpm.ru (M.P.); SKoretskiy@gnicpm.ru (S.K.); kharlapmaria@yahoo.com (M.K.); ODrapkina@gnicpm.ru (O.D.); 2 Erich and Hanna Klessmann Institute, Heart and Diabetes Center NRW, University Hospital of the Ruhr-University Bochum, Georgstrasse 11, 32545 Bad Oeynhausen, Germany; gpohl@hdz-nrw.de (G.M.P.); hmilting@hdz-nrw.de (H.M.); 3 All-Russia Research Institute of Agricultural Biotechnology, Timiryazevskaya Street, 42, 127550 Moscow, Russia; 4 Faculty of Bioengineering and Bioinformatics, Lomonosov Moscow State University, Lomonosovsky Prospect 27, Building 10, 119991 Moscow, Russia; 5 Medical Research and Educational Center, Lomonosov Moscow State University, Lomonosovsky Prospect 27, Building 10, 119991 Moscow, Russia; elena_mershina@mail.ru (E.M.); vsini@mail.ru (V.S.); 6 National Medical Research Center for Children’s Health, Lomonosovsky Prospect 2, Building 1, 119991 Moscow, Russia; basargina@nczd.ru (E.B.); gandaeva@nczd.ru (L.G.); woowka@mail.ru (V.B.); 7 National Medical Research Center for Cardiology, 3-ya Cherepkovskaya Street, 15A, 121552 Moscow, Russia; prof.boytsov@gmail.com

**Keywords:** cardiomyopathy, desmoglein-2, *DSG2*, left ventricular non-compaction cardiomyopathy, cardiovascular genetics, desmosomes, *TBX20*, dilated cardiomyopathy

## Abstract

Left ventricular non-compaction cardiomyopathy (LVNC) is a rare heart disease, with or without left ventricular dysfunction, which is characterized by a two-layer structure of the myocardium and an increased number of trabeculae. The study of familial forms of LVNC is helpful for risk prediction and genetic counseling of relatives. Here, we present a family consisting of three members with LVNC. Using a next-generation sequencing approach a combination of two (likely) pathogenic nonsense mutations *DSG2*-p.S363X and *TBX20*-p.D278X was identified in all three patients. *TBX20* encodes the cardiac T-box transcription factor 20. *DSG2* encodes desmoglein–2, which is part of the cardiac desmosomes and belongs to the cadherin family. Since the identified nonsense variant (*DSG2*-p.S363X) is localized in the extracellular domain of *DSG2*, we performed in vitro cell transfection experiments. These experiments revealed the absence of truncated *DSG2* at the plasma membrane, supporting the pathogenic relevance of *DSG2*-p.S363X. In conclusion, we suggest that in the future, these findings might be helpful for genetic screening and counseling of patients with LVNC.

## 1. Introduction

Left ventricular non-compaction cardiomyopathy (LVNC) is a rare structural cardiomyopathy, with or without left ventricular (LV) dysfunction, which is characterized by a two-layer structure of the myocardium in combination with an increased number of trabeculae [1]. The clinical presentation of LVNC is extremely diverse, ranging from asymptomatic cases to severe heart failure leading to life-threatening cardiac arrhythmias, sudden cardiac death, or thromboembolic complications [2,3]. Interestingly, in some patients with hypertrophic or dilated cardiomyopathies (DCM), a non-compaction phenotype can be found, raising the question whether LVNC is a distinct cardiomyopathy or a subtype. However, familial and sporadic cases of LVNC have also been previously reported [4]. Investigation of familial/genetic forms of cardiomyopathies might be helpful for risk prediction and genetic counseling of relatives [5]. Currently, mutations in more than 17 different genes have been described for LVNC [6]. From different cohorts of pediatric and/or adult patients, it can be estimated that 20–50% of LVNC patients have a genetic etiology [7,8], which may indicate there are yet undescribed genes associated with the development of LVNC.

Here, we present a family consisting of three members with LVNC, where we identified a combination of two (likely) pathogenic nonsense mutations in *TBX20* and *DSG2* genes (NP_001934.2:*DSG2*-p.S363X, NP_001071121.1:*TBX20*-p.D278X). We screened the genomic DNA of all available family members showing a cosegregation of both mutations within the family. *TBX20* encodes the cardiac T-box transcription factor 20 (OMIM, #606061). Mutations in *TBX20* are associated with congenital heart diseases, leading to atrial septal defects and DCM [9]. *DSG2* encodes desmoglein-2, which is part of the cardiac desmosomes and belongs to the cadherin family. Previously, mutations in the *DSG2* gene were associated with arrhythmogenic cardiomyopathy (ACM) [10] and DCM [11] but not LVNC. DSG2 is a type I transmembrane protein and consists of an extracellular domain mediating the protein–protein interactions with the neighboring cardiomyocytes. The cytoplasmic domain connects the desmosomes to the intermediate filaments [12]. Since the identified nonsense variant (*DSG2*-p.S363X) is localized in the extracellular part of DSG2, we performed in vitro cell transfection experiments. These experiments revealed the absence of truncated DSG2 at the plasma membrane, supporting the pathogenic relevance of *DSG2*-p.S363X. In summary, our study demonstrates that a combination of *TBX20* and *DSG2* mutations might be associated with a genetic form of LVNC. In the future, we therefore suggest that both genes are included in genetic analysis of further cases with LVNC.

## 2. Results

### 2.1. Clinical Investigations

**Figure 1:**
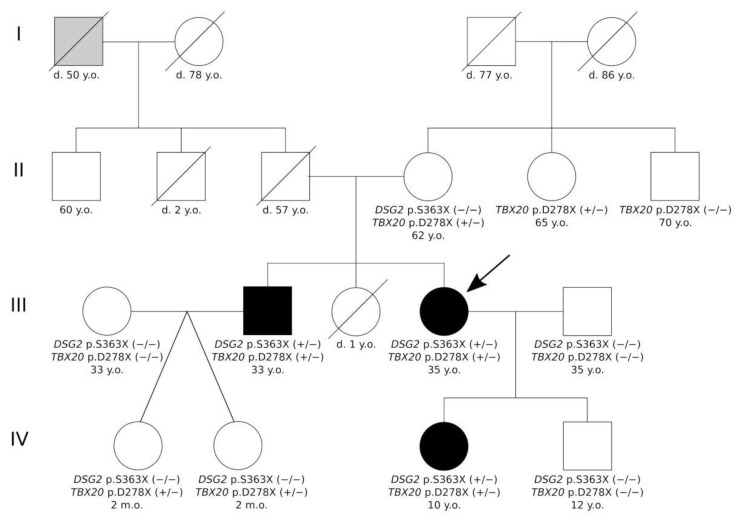
Pedigree of the described family. Circles represent females, squares males. Black-filled symbols indicate left ventricular non-compaction cardiomyopathy phenotype, grey symbols indicate members with an unknown type of cardiomyopathy, and white symbols indicate healthy family members. Backslashes indicate deceased members. +/− represent heterozygous and −/− wild type sequences for the indicated gene. The index patient (III-4) is marked with an arrow. d. = dead, m.o. = months old, y.o. = years old.

**Figure 2:**
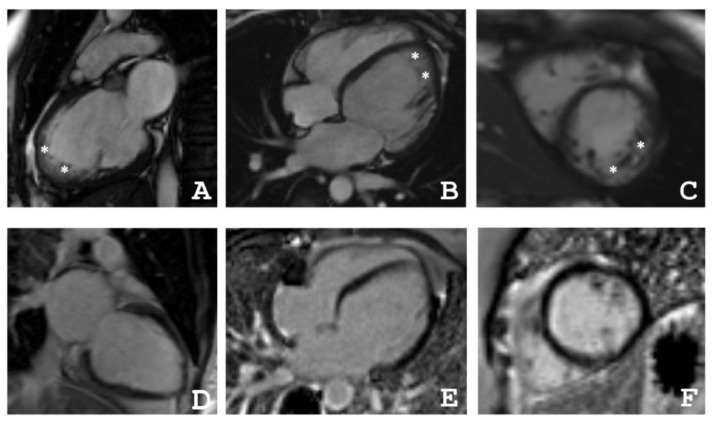
(**A**–**C**) Proband’s (III-4) cardiac magnetic resonance imaging in movie mode, steady-state free-precession (SSFP) sequence: (**A**) long axis 2–chamber projection, (**B**) long axis 4–chamber projection, (**C**) short axis at the level of the apical segments, * indicates a layer of non-compacted myocardium, (**D**–**F**) delayed contrast. Inversion recovery sequence with suppression of the signal from the myocardium. No areas of contrast, which indicates the absence of areas of intramyocardial fibrosis, scarring, and inflammatory myocardial damage.

The index patient of the described family (III-4, Figure 1) is currently a 35-year-old, normosthenic female. Tachycardia and shortness of breath during physical activity were diagnosed as a pupil. At the age of 21, during her first pregnancy, shortness of breath reoccurred. At the age of 24, during the second pregnancy, due to complaints of shortness of breath, an echocardiography test was performed, which showed LV dilatation and signs of cardiomyopathy. Therefore, she was treated with betaxalol. At the age of 30, she was infected with the flu and in the following she had palpitations, chest pain, and present shortness of breath. Two years later, she was examined in the National Research Center for Therapy and Preventive Medicine (Moscow, Russia). In blood tests, all indicators were within normal values, and brain natriuretic peptide (BNP) was 140 pg/mL. Electrocardiogram (ECG) shows sinus rhythm and left bundle branch block (LBBB). According to the Holter monitoring electrocardiogram (HM-ECG) (Bisoprolol 5 mg) she had sinus rhythm with a heart rate between 49–72–141 per min without any interruptions. Echocardiography revealed an end diastolic diameter (EDD) of 6.2 cm, an ejection fraction (EF) of 46%, and apical hypokinesia. She fulfilled the LNVC diagnostic criteria suggested by Jenni, Chin, Stollberger (see Table 1 and Table 2) [13,14,15]. In the magnetic resonance imaging (MRI), a non-compaction cardiomyopathy with dilatation of the LV without signs of myocarditis was present (Figure 2). Therapy for chronic heart failure was then started (ACE inhibitors, betablockers, mineralocorticoid receptor antagonist). Despite therapy with dynamic follow-up, EF decreased to 44%, BNP increased to 300 pg/mL, and shortness of breath and general weakness developed.

**Figure 3:**
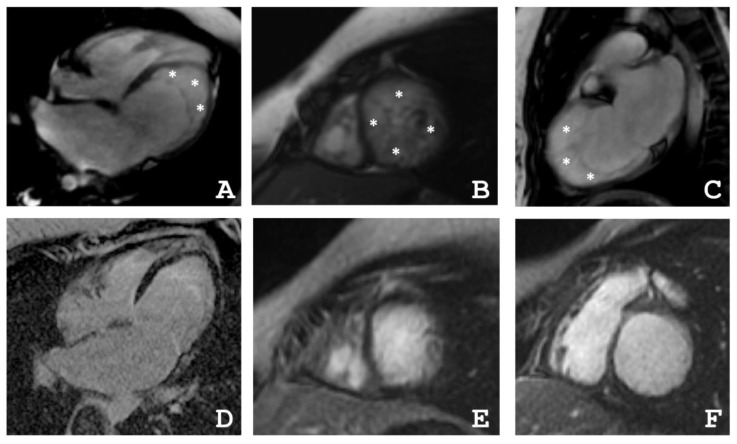
(**A**–**C**) Proband’s daughter (IV-3) cardiac magnetic resonance imaging in movie mode, SSFP sequence: (**A**) long axis 4–chamber projection, (**B**) short axis at the level of the apical segments, (**C**) long axis 2–chamber projection, * indicates the layer of non-compact myocardium, (**D**,**F**) delayed contrast. Inversion recovery sequence with suppression of the signal from the myocardium: (**D**) long axis 4–chamber projection, (**E**) short axis at the level of apical segments, (**F**) short axis at the level of basal segments. There were no areas of contrast, which indicates the absence of areas of intramyocardial fibrosis, scarring, and inflammatory myocardial damage.

The proband’s daughter (IV-3) is currently 10 years old. At the age of six, an increased LV size in combination with signs of LVNC were detected during routine echocardiography. After that, she was hospitalized in the National Research Center of Childhood (Moscow, Russia). According to echocardiography there were a small expansion of the LV (41/28 mm), of the left atrium (LA, 27 × 34 mm), and an EF of 60%. Signs of diastolic dysfunction of the restrictive type were observed, as well as non-compaction myocardium of the apical and middle segments according to the criteria suggested by Jenni et al. [14]. Cardiac MRI revealed non-compaction myocardium in the apical region with entry to the middle segment of the LV, increased LA, increased end diastolic volume (EDV), and end systolic volume (ESV) of the LV. In therapy ACE inhibitors, beta-blockers and metabolic therapy were used. During therapy, the condition remained stable, sometimes disturbed by an increased heart rate. Second hospitalization was at the age of eight years. According to HM–ECG, ventricular arrhythmia was excluded. Echocardiography parameters were LVED 41/28 mm, LA 27 × 34 mm, and EF 48%. BNP was 1531 pg/mL. The last clinical investigation was done at the age of nine. According to HM–ECG, the heart rate was within the reference and a transient delta wave was observed. Cardiac MRI revealed non-compaction myocardium with an EF of 48% (Figure 3). Fibrotic and inflammatory changes in the myocardium were not detected (Figure 3 and Table 3). Therapy was continued and dynamic clinical monitoring was performed. The patient’s health is currently stable and there are no clinical symptoms of heart failure.

**Figure 4:**
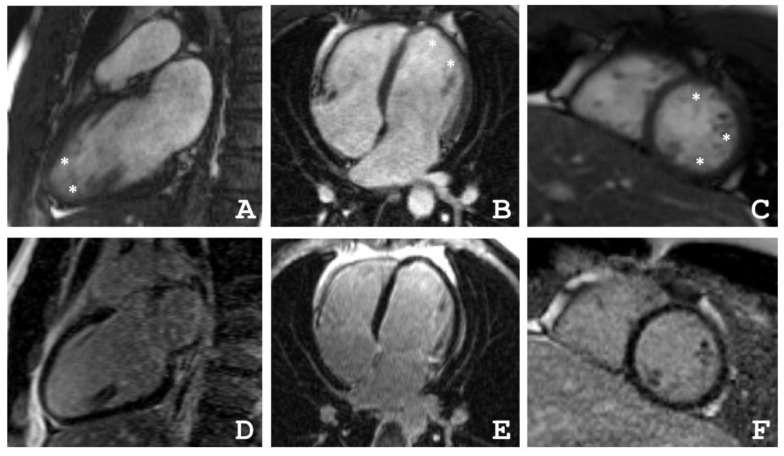
(**A**–**C**) Cardiac magnetic resonance imaging of the proband’s brother (III-2) in movie mode, SSFP sequence: (**A**) long axis 2-chamber projection, (**B**) long axis 4-chamber projection, (**C**) short axis at the level of apical segments, * indicates the layer of non-compacted myocardium, (**D**–**F**) delayed contrast. Inversion recovery sequence with suppression of the signal from the myocardium. There were no areas of contrast, which indicates the absence of areas of intra-myocardial fibrosis, scarring, and inflammatory myocardial damage.

The proband’s brother is a 33-year-old, normosthenic man (III-2, Figure 1). An ambulatory examination was carried out in respect to the identified disease of the sister. Echocardiography showed signs of non-compacted myocardium (Jenni criteria [14]). Both heart chambers were not dilated and diffuse hypokinesia was found. HM–ECG showed normal sinus rhythm. Cardiac MRI with gadolinium suggested LVNC (Figure 4). There were no signs of myocarditis.

The heart chambers of the proband’s mother (II-4, 62 years) were not dilated (EDD 5.2 cm, EDV 128 mL, EF 64%, diastolic dysfunction 1 type). HM–ECG showed normal sinus rhythm without ventricular arrhythmia. According to the patients’ data II-5 and II-6 had hypertension. III-1 and IV-1 were healthy. IV-2 had an atrial septal defect and hypertrabeculation on the apex.

### 2.2. Genetic Analysis

**Figure 5:**
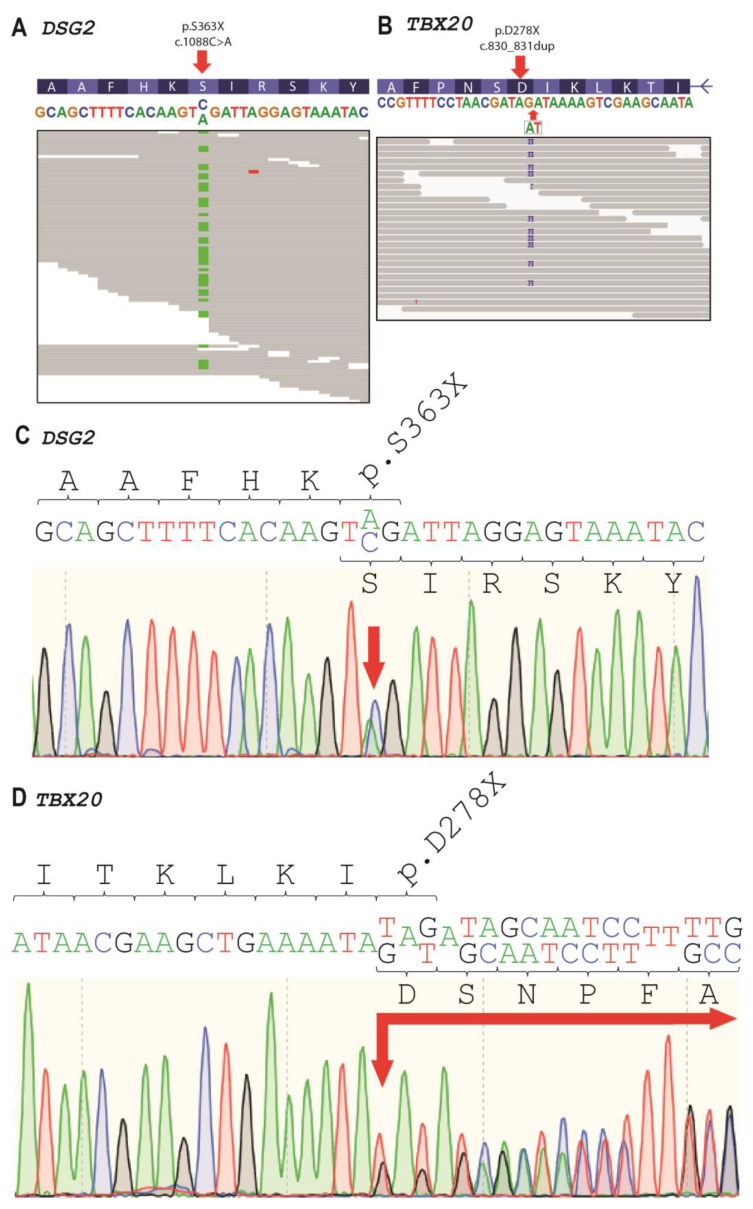
Genetic analysis of index patient (III-4). (**A**,**B**) Integrated genome view of *DSG2*-p.S363X and *TBX20*-p.D278X, respectively. (**C**,**D**) Electropherograms of *DSG2*-p.S363X and *TBX20*-p.D278X.

We performed genetic analysis with a whole-exome sequencing approach for the index patient III-4 and her relatives II-4, III-2, and IV-3. In the index patient, we identified six rare variants with a minor allele frequency (MAF) <0.001 (Table 3). One putative pathogenic heterozygous nonsense mutation in exon 9 of *DSG2* (hg19:chr18:29111023, NM_001943.5:c.1088C>A, NP_001934.2:p.S363X, rs751527714) was found (Figure 5A). In addition, we identified a second likely pathogenic *TBX20* nonsense variant (hg19::chr7:35271174, NM_001077653.2:c.830_831dup, NP_001071121.1:p.D278X) and four further missense variants with unknown significance (*SCN5A*, *TGFB3*, and two in *TTN*) (Figure 5B). Both variants were verified by Sanger sequencing (Figure 5C,D). *DSG2*-p.S363X was verified for III-2, III-4, and IV-3 (+/−) and *TBX20*-p.D278X variant was verified for II-4, II-5, III-2, III-4, IV-1, IV-2, and IV-3 (+/−) (Figure 1). 

### 2.3. Cell Culture Experiments

**Figure 6:**
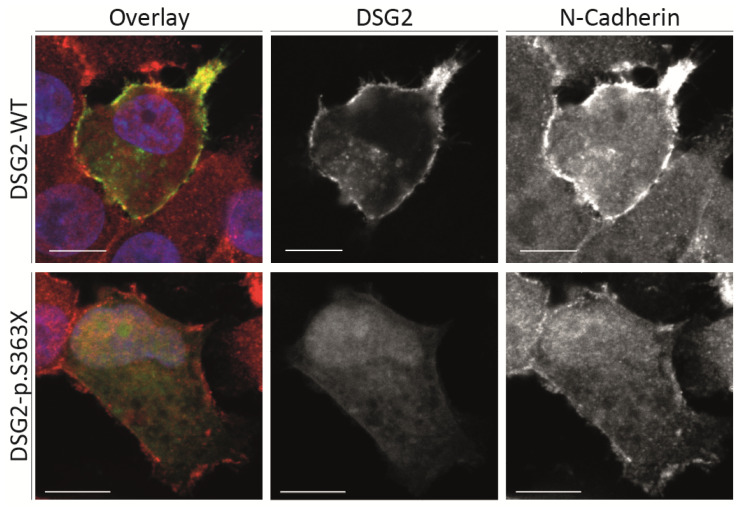
Cell transfection experiments reveal absence of truncated DSG2 at the plasma membrane. HT-1080 cells were transfected with pEYFP–N1–DSG2 (WT = wild-type) or the truncated pEYFP–N1–DSG2–p.S363X plasmids. The fluorescence signal of DSG2-EYFP is shown in yellow. N-Cadherin was costained using primary and Cy3-conjugated secondary antibodies and is shown in red. DAPI was used for staining of the nuclei and is shown in blue. Scale bars represent 10 µm. Of note, the truncated form of DSG2 is not localized at the plasma membrane.

Because p.S363X affects the extracellular domain of DSG2, we cloned this truncated form into the plasmid pEYFP-N1 and performed cell transfection experiments using HT-1080 cells. This cell line expresses endogenous desmosomal proteins and is therefore frequently used for functional analysis of desmosomal defects [19]. These cell transfection experiments revealed that the truncated form of DSG2 is unable to localize at the plasma membrane like the wild-type form (Figure 6). Only weak intracellular fluorescence signals could be detected in case of the mutant truncated DSG2. Since DSG2 is involved in cell–cell adhesion, these experiments supporting the pathogenicity of p.S363X and a loss-of function pathomechanism can be suggested.

## 3. Discussion

Currently, mutations in about 30 different genes have been described in patients with LVNC [6,20]. The majority of these genes encode for cytoskeletal proteins. However, in specific cases, the causative mutation cannot be identified, indicating that further genes might be involved in LVNC. Here, we present a three-generation family, where we identified two nonsense mutations in *DSG2* and *TBX20* cosegregating with LVNC within the family. Both variants can be classified according to the American College of Medical Genetics and Genomics (ACMG) guidelines as (likely) pathogenic mutations. *DSG2*-p.S363X is a null variant, and previously, it has been shown that DSG2 deficiency causes cardiomyopathy in mice and humans [21,22] (very strong criterion, PVS1, ACMG guidelines). Our functional analysis demonstrates in addition a damaging effect of this truncation mutation (strong criterion, PS3, ACMG guidelines). In addition, three moderate criteria (PM1, PM2, PM4, ACMG guidelines are fulfilled), because this mutation is absent in controls, changes the protein length and is localized in the extracellular domain, which is a hotspot for damaging cardiomyopathy-associated mutations. Furthermore, one supporting criterion is fulfilled by cosegregation of this mutation within the described family. According to the ACMG guidelines [23], *DSG2*-p.S363X has to be categorized as a pathogenic mutation. DSG2 belongs to the cadherin family and is localized in the cardiac desmosomes at the intercalated discs (IDs), mediating cardiomyocyte adhesion by calcium-dependent dimerization [24]. Desmosomal cadherins connect neighboring cells through homo- and heterophilic interactions in trans [25,26]. Loss of function mutations in *DSG2* affect cardiomyocyte cohesion, force transduction, and calcium processing [27]. Of note, a large number of studies have shown an association of mutations in *DSG2* and arrhythmogenic right ventricular cardiomyopathy (ARVC) [21,28,29]. However, other studies proved the relationship between the development of DCM and mutations in *DSG2* [11,30]. In 2008, Posch et al. found several variants in *DSG2* in patients with DCM [30]. A total of 73 patients were examined and sequenced with an assessment of the prevalence of missense variants in the control group. There were two missense variants of *DSG2* (p.V55M and p.V919G) that showed segregation with DCM in the family pedigree. Subsequent analysis of 538 patients with idiopathic DCM and 617 consecutive controls resulted in the identification of 13 *DSG2*-p.V55M carriers with DCM. In 2017, Kessler et al. showed that in pediatric DCM, most ID proteins follow similar spatio-temporal changes in localization as in controls. Endocardial and transmural fibrosis was increased in all pediatric DCM patients compared to age-matched controls [11]. It is worth noting that in our case, patients did not have myocardial fibrosis according to the cardiac MRI, which is atypical for patients with mutations in *DSG2*.

Despite the fact that the execution of the cell-based experimental study suggests that particular truncation/mutation *DSG2*-p.S363X prevent protein secretion in the experimental cell line, we cannot completely exclude the influence of allelic compensation of the wild-type allele of the *DSG2* protein, which is one of the study limitations.

In this case, we demonstrated for the first time a family with LVNC with a nonsense variant in the *DSG2* gene. Functional analysis revealed the absence of the truncated *DSG2* at the plasma membrane, supporting its pathogenicity. Currently, a genetic association between *DSG2* and LVNC has not been described in the literature yet. *DSG2* is one of the typical ARVC-associated genes. Despite the presence of the *DSG2* variant, there are no data for the presence of life-threatening ventricular arrhythmias and structural changes of the right ventricle (RV) in our patients. Mutations in *PKP2*, encoding plakophilin-2, which is a cytoplasmic binding partner of *DSG2*, are common for ARVC, but recently, a homozygous total deletion of *PKP2* was found in two siblings with a severe form of LVNC [31]. Therefore, it might be suggested that there is a genetic overlap of LVNC and ARVC.

Besides, the described LVNC patients (III-2, III-4, and IV-3, Figure 1) carry a second nonsense variant in *TBX20*. *TBX20* and *DSG2* are localized in humans on chromosome 7 and 18, respectively. *TBX20* encodes a transcription factor, which is involved in embryonic cardiac development and organogenesis (for a review, see [32]). Mutations in *TBX20* cause a complex and broad spectrum of heart defects, including septal defects, DCM, and arrhythmia [31]. Recently, Kodo et al. showed that a nonsense mutation in *TBX20* can also cause LVNC [33]. Functional analyses using induced pluripotent stem cell-derived cardiomyocytes (iPSC–CMs) revealed abnormal activation of the transforming growth factor beta (TGFβ) pathway [33]. Although we provide no functional data on *TBX20*-p.D278X, it can therefore be suggested based on different ACMG criteria that this mutation is likely pathogenic.

In addition to two (likely) pathogenic mutations in the *DSG2* and *TBX20* genes, we identified four rare missense variants in the *SCN5A*, *TTN*, and *TGF* genes in the proband and his relatives, but the relationship of these variants with the development of LVNC in this family seems unlikely to us. The variant *SCN5A*-p.R1511L can be classified according to the ACMG guidelines as a variant of unknown significance (VUS) (evidence of pathogenicity: PM2, PP3; evidence of benign impact: BS3, BS4). This variant has not been previously described in patients with cardiomyopathies, has no segregation in affected members of the family, and functional analysis has showed no damaging effect on protein function [34]. The variants *TTN*-p.R24089C and *TTN*-p.I13777T can be classified according to the ACMG guidelines as VUS (evidence of pathogenicity: PM2; evidence of benign impact: BS4, BP1). These variants have not been previously described in patients with cardiomyopathies, have no segregation in the affected members of the family, and are localized in a gene for which primarily truncating variants are known to cause the disease. The variant *TGFB3*-p.G262V can be classified according to the ACMG guidelines as VUS (evidence of pathogenicity: PM2, PP3; evidence of benign impact: none). This variant has been previously described only in one patient with arrhythmogenic right ventricular dysplasia in Clinvar (637043) without additional evidence of pathogenicity.

Presumably, both nonsense mutations in *DSG2* and *TBX20* contribute together to the LVNC phenotype in the affected patients, while two adult members of the family (II-4 and II-5, Figure 1) with the single mutation *TBX20*-p.D278X did not have signs of LVNC, indicating modifying effects of this mutation.

## 4. Materials and Methods

### 4.1. Clinical Description of the Patients

Three generations of the family with LVNC are presented. Family members underwent clinical examination, which included blood sample collection, biochemical and general examination, electrocardiography using 24-h HM-ECG, cardiac MRI, and echocardiography with contrast. Echocardiography and MRI imaging criteria were applied as previously suggested by Jenni et al. [14] and by Petersen et al. [18]. Our study was performed in accordance with the Declaration of Helsinki in its present form [35] and was approved by the Institutional Review Boards of the National Research Center for Therapy and Preventive Medicine (Moscow, Russia). Every participant and/or their legal representative gave their written informed consent to be involved in this study. 

### 4.2. Cardiac Magnetic Resonance Imaging

Cardiac MRI was performed with a 1.5-T imager (Avanto, Siemens, Munich, Germany) using a standard protocol. Breath-hold cine MRI was performed using ECG-gated segmented true fast imaging with steady-state free-precession (SSFP). Cine MRI was acquired in long-axis and short-axis planes covering the whole LV and RV. Late gadolinium enhancement images were acquired in the same planes 15 min after IV injection of the gadolinium contrast agent (Gd–DTPA–BMA, Omniscan, GE Healthcare, Marlborough, MA, USA) in a dose of 0.15 mmol/kg using inversion-recovery turbo fast-low angle shot (FLASH) pulse sequence.

### 4.3. Molecular Genetic Analysis 

DNA was isolated using the QIAamp DNA Blood Mini Kit (Qiagen, Hilden, Germany). DNA concentrations were determined on a Qubit 4.0 fluorimeter (Thermo Fisher Scientific, Waltham, MA, USA). Exome libraries were performed according to the IDT-Illumina TruSeq DNA Exome protocol (Illumina, San Diego, CA, USA). Next-generation sequencing was carried out on a Nextseq 550 (Illumina, San Diego, CA, USA). All stages of sequencing were carried out according to the manufacturer’s protocols. Variants with MAF <0.001 in 188 cardiomyopathy-associated genes (see Appendix A) were analyzed [21]. For clinical interpretation, genetic variants with frequencies in the gnomAD database of <0.5% were selected. Evaluation of the pathogenicity of the variants was carried out in accordance with the recommendations of ACMG [23]. For verification by Sanger sequencing, the following oligonucleotides were used: for *DSG2*-p.S363X 5′-AGTTGGACTATTCAGTGCTGCT-3′ and 5′-ACACGTGTCACTCTCTCTTACC-3′ (PCR product size—426 bp) and for *TBX20*-p.D278X 5′-GTACAAGGAATGGGGTGCAGA-3′ and 5′-TTTCCACCCTTCTCAGGACAC-3′ (PCR product size—350 bp). PCRs were performed in 20 μL of a mixture containing 0.2 mM of each nucleotide, 1× PCR buffer, 20 ng of the genomic DNA, 10 ng of each primer, and 2.5 U of DNA polymerase. Amplification was performed on a GeneAmp PCR System 9700 thermocycler (Thermo Fisher Scientific, Waltham, MA, USA) with the following parameters: 95 °C—300 s; 30 cycles: 95 °C—30 s, 62 °C—30 s, 72 °C—30 s; 72 °C —600 s. Before the Sanger reaction, the obtained amplicons were purified using ExoSAP-IT (Affymetrix, Santa Clara, CA, USA) according to the manufacturer’s protocol. The nucleotide sequences of PCR products were determined using the ABI PRISM BigDye Terminator reagent kit v. 3.1 (Thermo Fisher Scientific, Waltham, MA, USA) followed by analyses of the reaction products on an automated DNA sequencer Applied Biosystem 3500 DNA Analyzer (Thermo Fisher Scientific, Waltham, MA, USA). We analyzed the genomic DNA of all available family members (Figure 1).

### 4.4. Plasmid Generation

The full-length cDNA of *DSG2* and the truncated form *DSG2*-p.S363X were amplified by PCR using Phusion polymerase (Thermo Fisher Scientific, Waltham, MA, USA). PCR products were ligated in-frame with enhanced yellow fluorescence protein (EYFP) via *Xho*I and *Sac*II (Thermo Fisher Scientific, Waltham, MA, USA) into pEYFP-N1 (TaKaRa, Kyoto, Japan). Afterwards, the ligation reactions were transformed into *E. coli* DH5α strain via heat shock (42 °C, 30 s) and transformed single colonies were obtained by kanamycin sulfate selection (50 µg/mL, 37 °C, overnight). The complete *DSG2* encoding sequences of both plasmids pEYFP–N1–DSG2 and pEYFP–N1–DSG2–p.S363X were verified by Sanger sequencing (Macrogen, Amsterdam, Netherlands). Both plasmids were prepared using endotoxin-free plasmid preparation kits according to the manufacturer’s instruction (Thermo Fisher Scientific, Waltham, MA, USA).

### 4.5. Cell Culture, Immunocytochemistry and Confocal Microscopy

HT-1080 cells (German Collection of Microorganisms and Cell Cultures, DSMZ, Brunswick, Germany) were cultured in Dulbecco’s Modified Eagle Medium (DMEM, Thermo Fisher Scientific, Waltham, MA, USA) supplemented with 10% fetal calf serum (Thermo Fisher Scientific, Waltham, MA, USA) and penicillin and streptomycin as previously described [36]. Lipofectamine 3000 (Thermo Fisher Scientific, Waltham, MA, USA) was used for cell transfection according to the manufacturer’s instruction. Then, 24 h after transfection, the cells were washed with phosphate buffered saline (PBS) and were fixed for 10 min at room temperature (RT) using 4% Histofix (Carl Roth, Karlsruhe, Germany). After several gently washing steps with PBS, the cells were permeabilized using 0.05% Triton X-100 (10 min, RT). N-cadherin as a transmembrane protein expressed in HT-1080 cells was stained as a control using anti N-cadherin antibodies (#610920, 1:100, 4 °C, overnight, BD Bioscience, San Jose, CA, USA) in combination with Cy3-conjugated anti mouse immunoglobulin antibodies (#111165003, 1:100, RT, 1 h, Jackson ImmunoResearch, Cambridge, UK). Then, 4′,6-diamidino-2-phenylindole (DAPI, 1 µg/mL, RT, 5 min) was used to costain the nuclei. After washing with PBS, confocal microscopy was performed using the TCS SP8 (Leica Microsystems, Wetzlar, Germany) to detect the fluorescence emission of EYFP, Cy3, and DAPI as previously described [37].

## 5. Conclusions

In conclusion, we identified two cosegregating nonsense mutations in *DSG2* and *TBX20*, which are (likely) pathogenic for LVNC in the described family. Therefore, we suggest that in the future, these findings might be helpful for genetic screening and counseling of patients with LVNC.

## Figures and Tables

**Table 1:** Magnetic resonance imaging data. Ratio of non-compacted/compact layers by segment.

Patient	The Ratio NC/C in Segments																
	1	2	3	4	5	6	7	8	9	10	11	12	13	14	15	16	17
III-2	0	0	0	0	0	1.1	4.0	0	0	1.6	2.1	3.5	0	0	1.4	0	0
III-4	1.2	0	0	0	1.2	2.2	1.1	0	0	3.8	3.0	3.0	4.9	3.3	3.5	2.8	5.9
IV-3	3.3	1	0	0	0	0	3.5	2.5	1.1	0	0	1	8.3	8.3	6.3	8.3	10.5

NC = non-compaction layer, C = compaction layer.

**Table 2:** Magnetic resonance imaging data.

Patient	EDV	EF	Grothoff, [16]				Jacquier, % [17]	Petersen, [18]
			Mass Index of NC, g/m²	NC/Myocardial mass, %	NC/C ≥ 3:1 in One Segment(1–3, 7–16)	NC/C ≥ 2:1 in 4–6th Segments		
III-2	77	31	12	20	+	−	20	+
III-4	82	55	8	14	+	+	16	+
IV-3	64	48	21	36	+	−	36	+

EDV = end diastolic volume, EF = ejection fraction, NC = non-compaction myocardial layer, C = compacted layer. + indicates presence of trait, − indicates absence of trait.

**Table 3:** List of rare variants (MAF < 0.001) identified in the family.

Gene	Genomic Coordinates (GRCh37)	cDNA Change	dbSNP	Kind of Mutation	Protein Change	MAF ^1^	ACMG Classification	II-4	III-2	III-4	IV-3
*DSG2*	18:29111023	NM_001943.5:c.1088C>A	rs751527714	nonsense	NP_001934.2:p.S363X	0.000006577	Pathogenic	−	+	+	+
*SCN5A*	3:38597154	NM_000335.5:c.4532G>T	rs368219299	missense	NP_000326.2:p.R1511L	0.00002630	VUS	+	+	+	−
*TTN*	2:179402474	NM_003319.4:c.72265C>T	rs143556947	missense	NP_003310.4:p.R24089C	0.00009206	VUS	−	−	+	−
*TTN*	2:179442717	NM_003319.4:c.41330T>C	rs368301580	missense	NP_003310.4:p.I13777T	0.00002630	VUS	−	−	+	−
*TGFB3*	14:76429800	NM_003239.5:c.785G>T	rs1595339233	missense	NP_003230.1:p.G262V	0.00001381	VUS	−	+	+	+
*TBX20*	7:35271174	NM_001077653.2: c.830_831dup	NA	nonsense	NP_001071121.1:p.D278X	Novel	Likely pathogenic	+	+	+	+

^1^ According to the Genome Aggregation Database (gnomAD), https://gnomad.broadinstitute.org/, 17 May 2021; ACMG = American College of Medical Genetics and Genomics, MAF = minor allele frequency, NA = not applicable, VUS = variant of unknown significance. + indicates presence of heterozygous variant, − indicates absence of heterozygous variant.

## Data Availability

The data used and/or analyzed during the current study are available from the corresponding authors on reasonable request.

## Appendix A

Genes associated with different cardiomyopathies: *AARS2, ABCC9, ACAD9, ACADVL, ACTA1, ACTA2, AGK, AGL, AGPAT2, ALMS1, ANK2, ATP5E, ATPAF2, BRAF, BSCL2, CALR3, CAV3, CBL, COA5, COQ2, COX15, COX6B1, CRELD1, CRYAB, CTF1, CTNNA3, DES, DLD, DNM1L, DOLK, DSC2, DSG2, ELN, EMD, EYA4, FAH, FHL1, FHL2, FHOD3, FKRP, FKTN, FLNA, FOXD4, FOXRED1, FXN, GAA, GATA4, GATA6, GATAD1, GFM1, GJA1, GJA5, GLB1, GNPTAB, GUSB, HFE, HRAS, ILK, JAG1, JPH2, JUP, KCNJ2, KCNJ8, KLF10, KRAS, LAMA2, LAMA4, LIAS, MAP2K1, MAP2K2, MRPL3, MRPS22, MTO1, MURC, MYH11, MYLK2, MYOM1, MYOT, MYOZ2, NEBL, NEXN, NRAS, OBSL1, PDHA1, PDLIM3, PHKA1, PITX2, PMM2, PRKAG2, PSEN1, PSEN2, RAF1, RBM20, SCO2, SGCA, SGCB, SGCD, SHOC2, SLC22A5, SLC25A3, SLC25A4, SMAD3, SOS1, SPHA, SPRED1, SURF1, SYNE1, SYNE2, TBX1, TBX20, TBX5, TCAP, TGFB3, TMEM43, TMEM70, TMPO, TRIM63, TSFM, TTR, TXNRD2, VCL, XK.*

Genes associated with LVNC: *ACTC1, ACTN2, AMPD1, ANKRD1, ARFGEF2, BAG3, CASQ2, CNBP, CSRP3, DMD, DMPK, DNAJC19, DSP, DTNA, EYA1, FBN2, FLNC, GBE1, GLA, HADHB, HBB, HCCS, HCN4, HMGCL, ITGA7, KCNH2, KCNQ1, LAMP2, LDB3, LMNA, MIB1, MLYCD, MMACHC, MYBPC3, MYH6, MYH7, MYH7B, MYL2, MYL3, MYPN, NKX2-5, NNT, NOTCH1, PKP2, PLEC, PLEKHM2, PLN, PMP22, PRDM16, PTPN11, RYR1, RYR2, SCN5A, SDHA, SDHD, SIX1, SIX5, TAZ, TFAP2A, TNNC1, TNNI3, TNNT2, TNNT3, TPM1, TTN, YWHAE.*
